# Quantitative phase imaging through an ultra-thin lensless fiber endoscope

**DOI:** 10.1038/s41377-022-00898-2

**Published:** 2022-07-05

**Authors:** Jiawei Sun, Jiachen Wu, Song Wu, Ruchi Goswami, Salvatore Girardo, Liangcai Cao, Jochen Guck, Nektarios Koukourakis, Juergen W. Czarske

**Affiliations:** 1 Laboratory of Measurement and Sensor System Technique (MST), TU Dresden, Helmholtzstrasse 18, 01069 Dresden, Germany; 2 Competence Center for Biomedical Computational Laser Systems (BIOLAS), TU Dresden, Dresden, Germany; 3 State Key Laboratory of Precision Measurement Technology and Instruments, Department of Precision Instruments, Tsinghua University, 100084 Beijing, China; 4 Institute for Integrative Nanosciences, IFW Dresden, Helmholtzstraße 20, 01069 Dresden, Germany; 5 Max Planck Institute for the Science of Light & Max-Planck-Zentrum für Physik und Medizin, 91058 Erlangen, Germany; 6 Cluster of Excellence Physics of Life, TU Dresden, Dresden, Germany; 7 Institute of Applied Physics, TU Dresden, Dresden, Germany

**Keywords:** Imaging and sensing, Biophotonics

## Abstract

Quantitative phase imaging (QPI) is a label-free technique providing both morphology and quantitative biophysical information in biomedicine. However, applying such a powerful technique to in vivo pathological diagnosis remains challenging. Multi-core fiber bundles (MCFs) enable ultra-thin probes for in vivo imaging, but current MCF imaging techniques are limited to amplitude imaging modalities. We demonstrate a computational lensless microendoscope that uses an ultra-thin bare MCF to perform quantitative phase imaging with microscale lateral resolution and nanoscale axial sensitivity of the optical path length. The incident complex light field at the measurement side is precisely reconstructed from the far-field speckle pattern at the detection side, enabling digital refocusing in a multi-layer sample without any mechanical movement. The accuracy of the quantitative phase reconstruction is validated by imaging the phase target and hydrogel beads through the MCF. With the proposed imaging modality, three-dimensional imaging of human cancer cells is achieved through the ultra-thin fiber endoscope, promising widespread clinical applications.

## Abstract (web summary)

Graphical abstract for Quantitative phase imaging through an ultra-thin lensless fiber endoscope.

## Graphical abstract

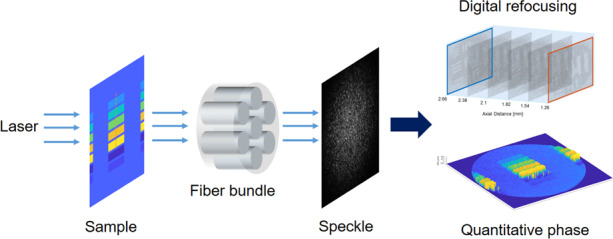

## Introduction

Quantitative phase imaging (QPI) is an effective and label-free method for cell and tissue imaging in biomedicine^1^. 3D images of transparent samples can be reconstructed with QPI in a non-invasive manner^2–10^, enabling nanoscale sensitivity to morphology and dynamics. Meanwhile, quantitative biophysical parameters such as refractive index^11,12^, dry mass^13,14^, matter density^15^, and skewness^16^ can be extracted from the quantitative phase shift, providing both morphological and quantitative biophysical information for digital pathology^17^. Recent research combining QPI with deep learning has been used for virtual staining^18,19^ and dynamic blood examination^20,21^, which was reported as a high throughput approach to detecting the SARS-CoV-2 virus^22^. On the other hand, current QPI methods are mostly based on bulky and expensive microscope platforms with limited working distance and penetration depth, which means invasive sampling or sectioning of diseased tissues or organs are required for pathological diagnosis^23,24^. Such invasive approaches limit the in vivo application of QPI in clinical diagnosis, especially in the early diagnosis of cancer and tumors.

**Fig. 1:**
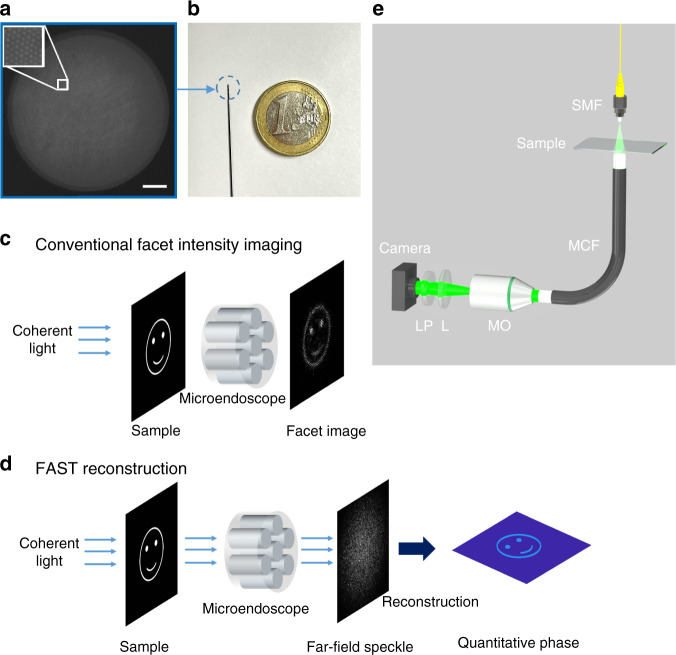
Lensless quantitative phase microendoscope: setup and concept. **a** Microscopic image of the tip facet of the 10,000 core fiber bundle with a diameter of 350 μm. Scale bar 50 μm. **b** Photo of the fiber bundle and a one euro coin for scale. **c** Conventional lensless microendoscopic imaging can only get the pixelated intensity information of the specimen, and the sample has to be very close to the fiber facet. **d** Quantitative phase and high-resolution amplitude images of the specimen can be reconstructed from the far-field speckle image. The sample can be placed far from the facet due to the digital focusing capability. **e** Experimental setup; SMF single-mode fiber, MO microscope objective, L achromatic lens, LP linear polarizer

In clinical diagnosis, endoscopes with diameters of a few millimeters are commonly used for in vivo imaging. Multi-core fiber bundle (MCF) is an ultra-thin fiber bundle of a few hundred micrometers consisting of thousands of single-mode fiber cores (Fig. 1a, b), and recent advances in MCF-based computational imaging demonstrate the great potential of fiber bundles to be the next generation microendoscopes with minimal invasiveness^25–27^. However, the phase information of the sample is lost due to the incoherent illumination. Despite computational methods that have been proposed to recover the 3D information of samples^28–30^, precise QPI via MCF with nanoscale sensitivity is still challenging. Coherent imaging is achieved via multi-mode fibers with transmission matrix measurement^31,32^ or wavefront shaping^33–38^, and similar approaches are also applied to MCF-based coherent imaging^39–45^. In practice, bulky and expensive optical systems with spatial light modulators and complicated calibration processes are still required, and the scanning-based imaging technique can be slow, inducing many limitations for clinical applications. Furthermore, an endoscope with nanoscale sensitivity of the optical path length is not yet reported, therefore, a simple and cost-effective 3D microendoscope with nanoscale sensitivity is highly demanded.

In this research, we found that the MCF can directly work as a phase encoder without a coded aperture^29^ at the measurement side, encoding the incident complex light field to a speckle pattern in the far-field at the detection side. We propose a novel computational approach named the far-field amplitude-only speckle transfer (FAST) method to decode the incident light field from the far-field speckles. Unlike conventional fiber facet imaging methods, where imaging resolution is limited by the core-to-core spacing (Fig. 1c), our approach enables 3D QPI reconstruction with nanoscale axial sensitivity of optical path length and lateral resolution up to 1 μm in the ideal case via direct recovery of the incident complex light field (Fig. 1d). We demonstrate a computational quantitative phase microendoscope (QPE) providing both morphological and quantitative biophysical information with a simple optical system (Fig. 1e), paving the path for in vivo clinical applications of the fiber bundles.

## Results

### Image reconstruction through the fiber bundle

In an MCF, the optical path length (OPL) varies for light traveling in different fiber cores, which results in a random phase distribution at the detection side for a plane wave illumination at the measurement side. The intrinsic OPL difference is stable when the fiber bundle is static in the measurement process. The phase shift induced by the sample can thus be reconstructed from intensity-only far-field speckles at the detection side.

**Fig. 2:**
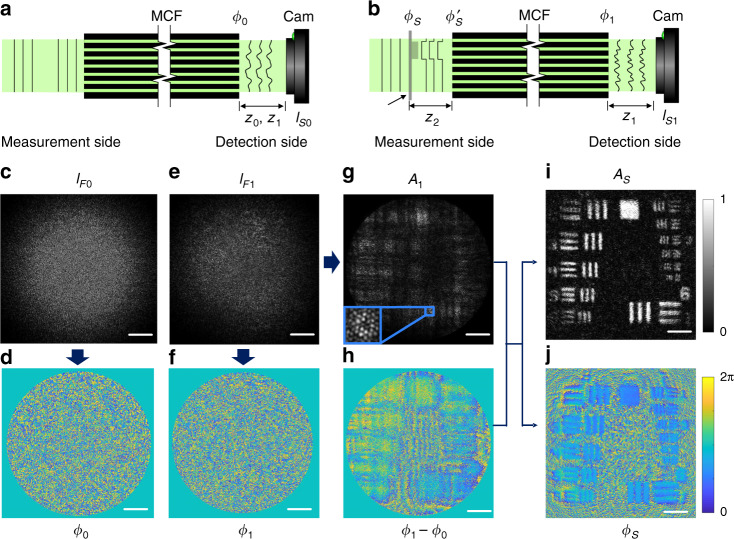
Working principle of the lensless quantitative phase microendoscope. **a** The phase of a coherent light source is distorted by the MCF at the detection side. **b** A USAF resolution test target is located 1.6 mm away from the facet. The far-field speckle image is captured on the camera. **c** Reference speckle image captured on the detection side with a point light source illumination on the measurement side. **d** Reconstructed reference phase image on the fiber facet. **e** Far-field speckle image of the test target. **f** Phase and **g** amplitude reconstruction on the fiber facet, which contains the light field information of the test target. **h** Retrieved phase image of the diffracted test target. Reconstructed **i** amplitude and **j** phase image of the 6th and 7th group elements of the test target on the focal plane. Scale bars 50 μm

The imaging principle and reconstruction process of the lensless quantitative phase microendoscope is demonstrated in Fig. 2. Initially, the MCF is illuminated by a collimated laser beam or a point light source for a reference measurement of the intrinsic OPL difference of fiber cores. Two far-field speckle patterns, which are *z*_0_ and *z*_1_ away from the fiber facet at the detection side, are magnified and projected on the camera at the detection side (Fig. 2a). The intrinsic phase shift of the MCF (Fig. 2d) induced by the OPL difference is reconstructed from the far-field speckles (Fig. 2c) with the FAST algorithm (see Supplementary materials).

A negative resolution test target, where only the pattern is transparent, is put 1.6 mm (*z*_2_) away from the facet as a sample at the measurement side (Fig. 2b). The speckle pattern *z*_1_away from the facet, which is the system response of the sample, is captured on the camera at the detection side (Fig. 2e). The phase *ϕ*_1_(*x*,*y*) and amplitude *A*_1_(*x*,*y*) information on the facet is reconstructed from the intensity-only far-field speckle. The phase of the sample is encoded at the detection side as shown in Fig. 2f due to the fiber core OPL difference. The original phase incident on the fiber bundle at the measurement side $\phi _s^\prime (x,y)$, which contains the phase information of the test target, can be decoded by the measured intrinsic phase shift *ϕ*_0_(*x*,*y*) as shown in Fig. 2h.

1 $$\phi _s^\prime = \phi _1 - \phi _0$$

On the other hand, the original amplitude information of the incident light field is maintained at the detection side as shown in Fig. 2g. Therefore, the incident light field on the measurement facet can be expressed as a complex field $E_s^\prime (x,y)$.

2 $$E_s^\prime = A_1 \cdot \exp (i\phi _s^\prime )$$

Hence, the incident light field is back-propagated numerically to the sample plane with the angular spectrum method^46^. The digital-focused amplitude and phase image of the test target is calculated from the propagated complex field. The 6 and 7 group elements of the test chart are resolved in both amplitude (Fig. 2i) and phase (Fig. 2j) reconstruction through the MCF. It can be noticed that the field of view is further extended beyond the size of the fiber facet with the FAST technique.

### Digital refocusing

**Fig. 3:**
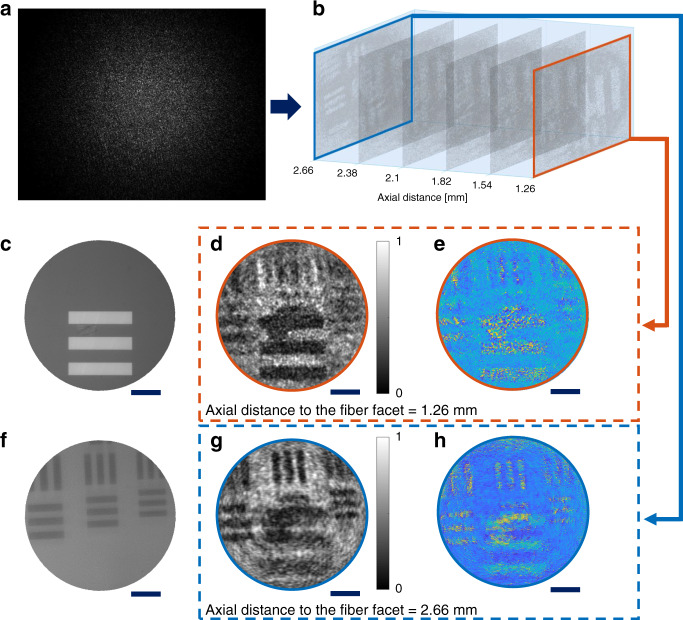
Reconstruction of a multi-layer target, which consists of two test targets located at 1.26 and 2.66 mm away from the facet at the measurement side. **a** Speckle pattern of the multi-layer target captured on the detection camera. **b** Reconstructed amplitude slides of the sample at different axial distances. **c** Image of the top layer from a bulky reflective microscope. **d**, **e** Reconstructed **d** amplitude and **e** phase image of the top layer, the linewidth is 22.1 μm. **f** Image of the bottom layer from a bulky reflective microscope. **g**, **h** Reconstructed **g** amplitude and **h** phase image of the bottom layer, the linewidths are 11.05, 9.84, and 8.77 μm respectively. Scale bars 50 μm

Two stacked positive resolution test targets, where the patterns are not transparent, are used to characterize the digital refocusing capability of the proposed microendoscope system. The axial distance between the patterns on the test targets is 1.4 mm, and the top layer is 1.26 mm away from the facet at the measurement side. On the detection side, the 3D information of the light field is stored in the far-field speckle (Fig. 3a). The complex field of the incident light at the measurement side is reconstructed from the proposed method. Hence, the slices at various axial distances are recovered from the complex field by numerical propagation (Fig. 3b). A reconstructed video demonstrating the digital focusing process is shown in Supplementary video V1.

Images of the stacked test targets at both layers can be also acquired with a bulky reflective microscope (Fig. 3c, f), but mechanical tuning of the sample position is required. In contrast, images of samples located at multiple axial distances (Fig. 3d, g) can be reconstructed from a speckle image captured at the detection side of the MCF. The phase information can also be recovered at different axial distances as shown in Fig. 3e, h. The lines on the test target are not transparent, which leads to the random phase distribution in the area of lines.

### Glass bead flow reconstruction

A reconstructed video demonstrating the glass bead flow in a microchannel which is imaged through the microendoscope is demonstrated in Supplementary video V2. The glass bead suspension is pumped into a microchannel constantly by a syringe. The channel is located 1 mm away from the fiber facet at the measurement side. The corresponding far-field speckles at the detection side are recorded on the camera at a frame rate of 10 frames per second, and all the frames are processed offline.

### Quantitative phase imaging reconstruction

Phase imaging can provide additional contrast in label-free microscopic imaging, and precise measurement of quantitative phase values can define the refractive index or thickness of biomedical samples. Due to the nanoscale sensitivity of the optical path length, the proposed system has the potential to further measure the height of nanoscale semiconductor structures.

**Fig. 4:**
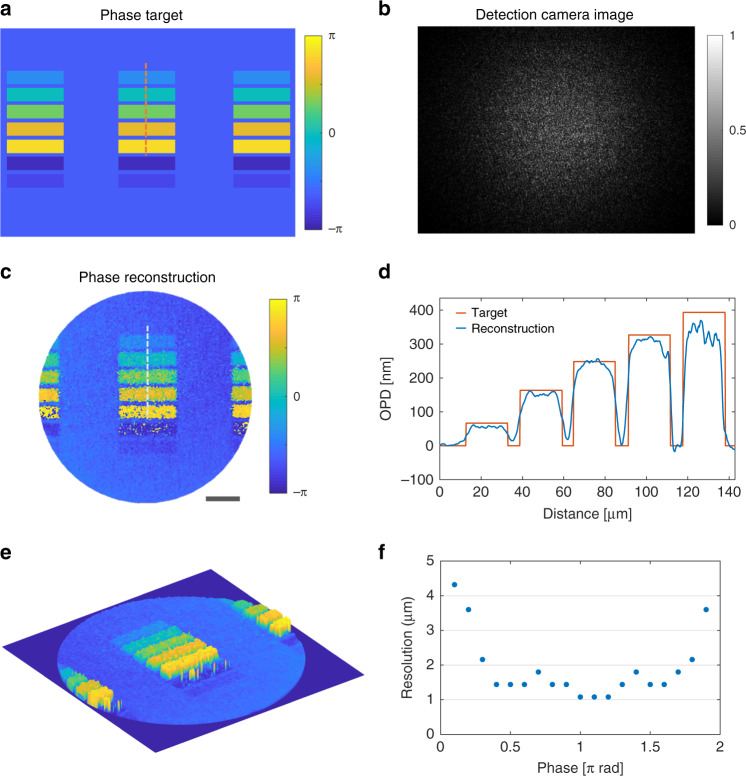
Quantitative phase imaging through the microendoscope via far-field speckle reconstruction. **a** The phase target is imaged on the distal side of the microendoscope. **b** The far-field speckle image of the phase target is captured on the camera. **c** Reconstructed phase image of the target from the speckle image. Scale bar 50 μm. **d** Quantitative optical path difference profile of the marked areas in (**a**, **c**). **e** 3D optical path difference map. **f** The measured lateral resolution of the system for different phase values. The measurement process is demonstrated in the Supplementary materials

A phase target shown in Fig. 4a is used to characterize the precision of the reconstructed phase from the MCF microendoscope. The phase target is projected on the MCF at the measurement side, and the corresponding system response on the detection camera is shown in Fig. 4b. The quantitative phase image is reconstructed from the speckle image with the FAST method. The phase tilt in the background is corrected numerically and a simulated phase mask with the same phase tilt is subtracted to correct the phase value in the background. The final quantitative phase reconstruction is demonstrated in Fig. 4c. Colors in the phase image represent different phase values, hence, the quantitative phase information is successfully recovered with the FAST reconstruction. A vital optical parameter—optical path difference (OPD), which correlates the refractive index and the thickness of the sample, can be calculated from the quantitative phase shift. A comparison of the calculated OPD between the original phase target and the phase reconstruction through the fiber bundle is demonstrated in Fig. 4d, characterizing the high fidelity of the quantitative phase reconstruction. The data colored in orange indicates the data sampled from the phase target and the blue represents the sampled data from the phase reconstruction. The size of the sample area is 142 μm in the longitudinal direction and 2μm in the lateral direction. The calculated 3D OPD map is demonstrated in Fig. 4e and Supplementary video V3.

To further characterize the resolution limit of the system, a program-controlled phase target with tunable phase value and size is implemented. A detailed explanation of the measurement process is demonstrated in supplementary materials. As shown in Fig. 4f, due to the relatively low signal-to-noise ratio (SNR) for the target with an absolute phase shift lower than 0.4π, the lateral resolution of the system ranges from 2 to 4.32 μm. Nevertheless, the lateral resolution of the phase reconstruction can reach up to 1 μm for the target with a higher phase shift. It has to be noted that such resolution is achieved with an ideal phase object with homogeneous refractive index distribution.

### 3D imaging of biomedical samples through the fiber endoscope

Both morphological and quantitative biomedical parameters can be extracted from an OPD image, indicating the great potential in biomedical applications of the quantitative phase microendoscope. The cell-like Polyacrylamide (PAAm) hydrogel bead has a spherical shape and homogeneous refractive index distribution^47^. This makes PAAm beads ideal for verifying the OPD measurement fidelity with the microendoscope for biomedical samples.

**Fig. 5:**
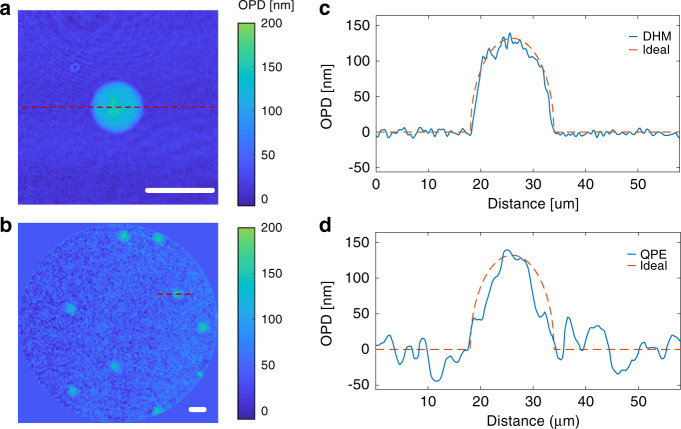
Quantitative phase imaging of micro-gel beads via microendoscope. Quantitative optical path difference (OPD) map of PAAm micro-gel beads acquired with **a** digital holographic microscope (DHM), **b** quantitative phase microendoscope (QPE). Scale bars 20 μm. **c**, **d** OPD distribution along the red marked lines in the maps on the left side. The dashed lines represent the ideal OPD distribution of the PAAm bead

A reference OPD measurement of the bead is done on a digital holographic microscope (DHM) (Fig. 5a). The average diameter of the beads is measured as 16.7 μm. PAAm beads in suspension are also resolved clearly in the reconstructed OPD map with the MCF-based microendoscope (Fig. 5b). Due to the ideal spherical shape and homogeneous refractive index distribution of the beads, the refractive index of the beads can be calculated precisely from the OPD (Eq. 3). The OPD distribution of the lines (marked in red in Fig. 5a, b) through the center of the bead are demonstrated as blue lines in Fig. 5c, d. The simulated OPD distribution of an ideal sphere with a refractive index difference of 0.008 to the background, demonstrated as orange dashed lines in Fig. 5c, d, fits the measured OPD distribution well. The refractive index of the medium (D-PBS) is determined as 1.335, hence the refractive index of the PAAm beads is measured as 1.343. Although the reconstructed OPD map from the microendoscope has a relatively lower spatial resolution and higher background noise, the quantitative OPD values of the beads are recovered correctly. This verifies the strength of the quantitative phase microendoscope to provide accurate OPD measurement at the single-cell level.

**Fig. 6:**
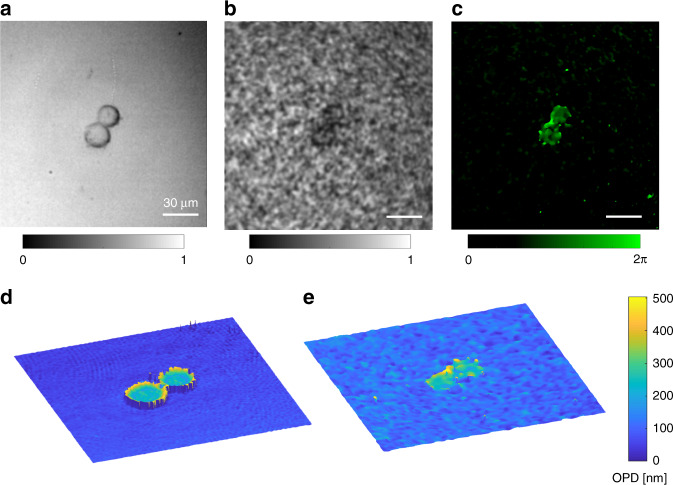
Quantitative phase imaging of cancer cell cytokinesis via microendoscope. **a** Intensity image of a HeLa cell in cytokinesis from a conventional reflective microscope. **b**, **c** Microendoscopic **b** amplitude and **c** phase reconstruction of the HeLa cell in cytokinesis from the far-field speckle. Scale bars 30 μm. **d**, **e** 3D OPD map of the HeLa cell measured from the **d** conventional digital holographic microscope and the **e** quantitative phase microendoscope

Human cancer cells are used to characterize the performance of the quantitative phase imaging for biological cells via microendoscope. The image of a HeLa cell in cytokinesis captured from a bulky reflective microscope is shown in Fig. 6a. The reconstructed amplitude image of the same cell through the microendoscope is demonstrated in Fig. 6b. The cell is still distinguishable from the background noise. The contrast is significantly improved in the reconstructed phase image (Fig. 6c). The cancer cell undergoing cytokinesis is clearly resolved by a microendoscope without labeling. It is visible in the phase reconstruction that the cell membranes of two daughter cells are not separated yet. The 3D OPD map of the HeLa cell is thus calculated from the phase shift measured by the quantitative phase microendoscope (see Fig. 6e and Supplementary video V5). A high-resolution OPD map of a similar HeLa cell in cytokinesis is reconstructed from the DHM as a reference measurement (see Fig. 6d and Supplementary video V4).

## Discussion

Our results demonstrate that the ultra-thin lensless MCF endoscope provides high-resolution QPI in hard-to-reach areas. The reconstructed OPD map demonstrated sufficient image quality for morphological evaluations of live human cancer cells. Vital cellular parameters such as cell volume^1^, dry mass^13,14^, and refractive index^2,48^ can also be extracted from the precise OPD map for cytopathology investigation and clinical diagnosis. The precise quantitative phase imaging performance can be hardly achieved using other endoscopes illuminated by incoherent light sources due to the wide spectrum of the light source. Digital holography is a common method for quantitative phase imaging^49,50^, however, digital holographic imaging with long MCFs requires complex optical systems and tedious optical alignment^44,51^. Our proposed FAST reconstruction method does not require digital holography, the precise amplitude and phase images of the sample can be recovered from an intensity-only speckle. The 2D image correlation between the amplitude reconstruction with the off-axis holography and the FAST method is 0.998 (Fig. S6). The quantitative phase of the sample is also precisely retrieved with a lower phase noise level in the background compared to the digital holographic reconstruction (Fig. S6e, f). Therefore, the holography-free lensless microendoscope based on FAST reconstruction provides sufficient reconstruction quality for both amplitude and phase images with a significantly simplified optical system.

Furthermore, the proposed method enables digital refocusing of the reconstructed complex light field to different depths, which significantly increases the depth of field of the lensless microendoscope to several millimeters and gives more degrees of freedom in sample examinations. The miniature lensless microendoscope with a diameter of 0.35 mm is so far the tiniest imaging probe with micrometer range lateral resolution and nanoscale axial sensitivity, paving the way to in vivo label-free detection with minimal invasiveness.

Different from our previously reported lensless endoscope based on 3D scanning imaging^41,42^, the current setup can achieve comparable lateral resolution and higher axial resolution from the numerical reconstruction of a far-field speckle pattern. It has to be noted that such a scanning-free imaging modality is highly desired for high throughput measurements and dynamic monitoring of samples because the image capturing speed can reach the maximum frame rate of the detection camera. Due to the simplicity of the proposed method, digital holography and wavefront shaping are not necessary, which leads to a compact and cost-effective system. In addition, compared to endoscopes implementing micro-lens or printed structures on fiber tips^27,29,30^, our system is built from a commercially off-the-shelf fiber bundle and common optical components, which is easily replicable for further applications.

The iterative process of the FAST reconstruction may raise concerns about computational time. Due to the complexity of the reference phase shift of the fiber bundle (Fig. 2d), two speckles at different axial distances are required as the input for the FAST reconstruction. The reconstruction is operated on MATLAB and takes about 8 min to calculate the reference phase shift (2560 × 1920 pixels) on a desktop computer (CPU, AMD Ryzen Threadripper 3960X) with GPU (NVIDIA TITAN RTX) acceleration. To reconstruct the further phase shift caused by the sample (Fig. 2d), only a single speckle image is required with the reference phase shift as the initial phase for the iteration, and it only takes about 24 s for a single reconstruction. Performing the reconstruction on multiple GPUs in a parallel pool can further improve the computational time, because the algorithm relies on 2D FFT. The stability of the system when bending the fiber bundle is a critical attribute for in vivo applications. Slight deformation of the fiber bundle after the reference measurement would lead to an additional global tilt of the image plane on the detection side^37,52^. The resulting tilt on the phase reconstruction, which is extracted from the background, can be further corrected numerically. Recently reported custom-designed twisted MCF, shows a performance independent of the fiber bending^53^, which would further increase the degree of freedom of a lensless microendoscope.

## Materials and methods

### Multi-core fiber bundle

A 40-cm-long fiber bundle (FIGH-350S, Fujikura, Japan) with around 10,000 cores is used in this work. The diameter of the fiber bundle is 350 μm. The average core diameter is 2μm and core-to-core spacing is 3.2 μm.

### Experimental setup

The experimental setup is shown in Fig. 1e. A 532 nm diode-pumped solid-state continuous-wave laser (Verdi, Coherent Inc., USA) is coupled into a single-mode fiber (460HP, Thorlabs, Germany), and the output beam from the single-mode fiber is used to illuminate the sample. The diffracted light field incident the MCF at the measurement side. On the detection side, a 10× microscope objective (0.25 NA; Plan Achromat Objective, Olympus) and an achromatic lens (*f* = 200 mm; Thorlabs, Germany) compose a 4-f system. Hence, the magnified far-field speckle can be projected on the detection camera (UI-3482LE, IDS GmbH, Germany). Due to the random birefringence of the fiber cores, a linear polarizer is placed in front of the camera (LPVISE100-A, Thorlabs, Germany) for capturing a linearly polarized light field.

### Speckle reconstruction algorithm

A specialized phase retrieval algorithm for the fiber bundle is implemented to reconstruct the phase on the fiber facet from the far-field speckle image. The iterative reconstruction process is demonstrated in Fig. S1 and explained in supplementary materials. The total variation minimization algorithm^54^ is implemented on the reconstructed amplitude images to reduce the speckle noises. On the other hand, a 2D median filter is applied to the reconstructed phase images to reduce the phase spikes.

### Optical path difference (OPD)

When a coherent light propagates through a homogeneous medium with a refractive index *n*, the OPL is defined as the product of the geometric traveling distance *d* of light. Therefore, the OPLs are different when the coherent light travels through mediums with different refractive indices *n*_0_, *n*_1_ at the same distance *d*, and the OPD is defined as

3 $${{{\mathrm{OPD}}}} = (n_1 - n_0)d$$

In experiments, the OPD can be measured from the phase shift Δ*ϕ* of a coherent light source that passes through mediums with different refractive indices

4 $${{{\mathrm{OPD}}}} = (\frac{{\Delta \phi }}{{2\pi }} + k)\lambda$$

where *k* is non-negative integers, *λ* is the wavelength of the light source. Hence, the phase shift also corresponds to the refractive index difference and the thickness of the medium.

5 $$\Delta \phi = \frac{{2\pi d}}{\lambda }(n_1 - n_0) + 2\pi k$$

### Phase target

The phase target shown in Fig. 4a is displayed on a spatial light modulator (PLUTO, Holoeye Photonics AG, Germany) and projected on the fiber facet at the measurement side.

### Microgel beads preparation

The polyacrylamide (PAAm) microgel beads functionalized with fluorescent dye were produced by using a microdroplet generation system and protocol described in a previous study^47^. The continuous phase was a fluorinated oil (HFE-7500, Ionic Liquids Technology, Germany) containing ammonium Kritox® surfactant, N,N,N’,N’-tetramethylethylenediamine (TEMED), and acrylic acid N-hydroxysuccinimide ester (Sigma-Aldrich Chemie GmbH, Germany). The dispersed phase was a pre-gel mixture of acrylamide, N,N’-methylenebis acrylamide, ammonium persulphate (Sigma-Aldrich Chemie GmbH, Germany) and Alexa Fluor® 488 Hydrazide (Thermo Fisher Scientific, Germany) dissolved in 10 mM Tris-buffer. The flow of the two phases was controlled by a pressure microfluidic controller (Fluigent MFCSTM-EX) and adjusted to obtain beads with a final diameter of about 16 µm, analyzed by bright-field microscopy. A ratio of the cross-linking agent to a monomer of 3.25% and a total monomer concentration of 9.9% resulted in beads with Young’s modulus of about 6kPa, measured by AFM indentation. The functionalized PAAm beads were washed and re-suspended in 1× PBS and stored at 4 °C until further use. To image the PAAm beads with the microendoscope, the beads are suspended in DPBS (Thermo Fisher, USA) and located 0.5 mm away from the measurement fiber facet.

### HeLa cell preparation

The stable HeLa cell line was kindly provided by the lab of Mariana Medina Sánchez (Leibniz Institute for Solid State and Materials Research). HeLa cells were cultured at 37 °C in a humidified atmosphere containing 5% CO_2_ in Dulbecco’s modified Eagle’s medium (DMEM) (Thermo Fisher, USA) supplemented with 10% (v/v) fetal bovine serum (FBS) (Thermo Fisher, USA), 100 U/mL penicillin, and 100 μg/mL streptomycin. HeLa cells were recovered and incubated for 2 weeks before use for spheroids culture. Equal amounts of HeLa cells (2 × 10^5^ cells resuspended in 4 mL) were added to 3.5 cm cell-repellent dishes (Greiner bio-one) after trypsinization and washing with PBS (Thermo Fisher, USA) for preparing spheroids with homogeneous sizes. After two days of maturation, spheroids were separated into different groups and incubated with related treatments. The culture medium was exchanged to DPBS (Thermo Fisher, USA) without phenol red before the measurement.

## Supplementary information

Supplementary Information

Supplementary video V1

Supplementary video V2

Supplementary video V3

Supplementary video V4

Supplementary video V5

## Data Availability

The Matlab code of the FAST algorithm is publicly available on Github at https://github.com/Jiawei-sn/FAST.
