# Supplementary material for: Quantitative phase imaging through an ultra-thin lensless fiber endoscope
Source: Light Sci Appl. 2022 Jul 5;11:204. doi: 10.1038/s41377-022-00898-2 (PMC9255502; doi:10.1038/s41377-022-00898-2)
Supplement: Supplementary file 1 — Supplementary Information [file 41377_2022_898_MOESM1_ESM.docx]

Supplementary Information for

**Quantitative phase imaging through an ultra-thin lensless fiber endoscope**

Jiawei Sun, Jiachen Wu, Song Wu, Ruchi Goswami, Salvatore Girardo, Liangcai Cao, Jochen Guck, Nektarios Koukourakis, and Juergen W. Czarske

*Corresponding author. Email: jiawei.sun@tu-dresden.de (J.S.);

nektarios.koukourakis@tu-dresden.de (N.K.);

juergen.czarske@tu-dresden.de (J.C.)

**This file includes:**

Supplementary Methods

Figs. S1 to S6

Supplementary video caption V1 to V6

References (1 to 8)

**Other Supplementary Materials for this manuscript include the following:**

Supplementary video V1 to V6

Supplementary Methods

**Numerical propagation**

For propagating the light field numerically, both amplitude $A(x,y)$ and phase information are required to form the complex light field $E(x,y)$ at the initial axial plane

Based on the angular spectrum method, after a propagation distance of $z$, the complex light field $E_{z}(x,y)$ can be calculated by the angular spectrum method as

$$E_{z}=\text{iFFT}\{\text{FFT}\{E\} e^{ jz\sqrt{(2\pi/\lambda)^{2}- (k_{x}^{2}+k_{y}^{2})}}\}.$$

where FFT and iFFT represent the fast Fourier transform and the inverse fast Fourier transform, $\lambda$ is the wavelength of the light,$k_{x}$,$k_{y}$are the spatial frequencies. The propagated amplitude $A_{z}(x,y)$ and phase can thus be calculated from the complex light field

**Far-field amplitude-only speckle transfer (FAST)**

We demonstrate the far-field amplitude-only speckle transfer (FAST) algorithm to reconstruct the random phase distribution on the facet from the far-field speckle images on the detection side. Common phase retrieval algorithm ^1–3^ is robust for recovering the phase information of simple objects, however, it is difficult to recover the random phase distribution of the 10,000 core of the fiber bundle due to its complexity. The algorithm stagnates near the closest local minimum when we try to recover the phase on the facet. Hence, an algorithm provides faster convergence and stronger constraint is demanded in this case. Inspired by the multi-distance phase retrieval ^4^, which is initially utilized for increasing the convergence speed of the algorithm, we use two far-field speckle images captured at two axial distances to recover the complex phase distribution on the facet. The principle of the phase retrieval process is demonstrated in Fig. S1.


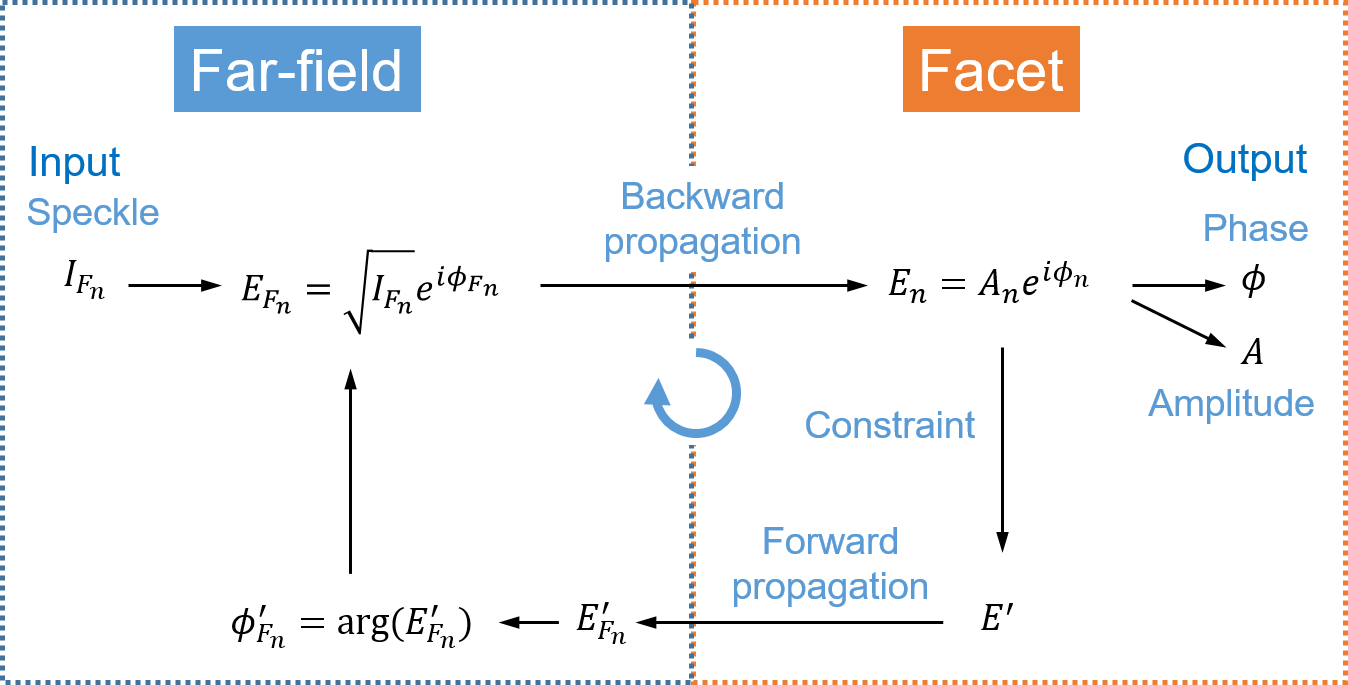


Fig. S1. Diagram of the iterative phase retrieval algorithm.

To be more specific, two input speckle intensity images $I_{F_{1}}(x,y)$, $I_{F_{2}}(x,y)$ (Fig. S2a) are converted to the amplitude $\sqrt{I_{F_{1}}(x,y)}$, $\sqrt{I_{F_{2}}(x,y)}$ of the complex light field in the far field. However, the phase of the complex field is still unknown, hence, random phases , are used to form the estimated far-field complex field $E_{F_{1}}(x,y)$, $E_{F_{2}}(x,y)$ at $z_{1}$, $z_{2}$.

Both complex fields are then back-propagated to the facet plane numerically and two estimated light fields on the facet plane are obtained as

$$E_{n}=\text{iFFT}\{\text{FFT}\{E_{F_{n}}\} e^{ -jz_{n}\sqrt{(2\pi/\lambda)^{2}- (k_{x}^{2}+k_{y}^{2})}}\}.$$

A binary mask $M(x,y)$(see Fig. S2d), which is obtained by thresholding the captured amplitude image at the facet plane (Fig. S2e), represents the morphology of the fiber bundle facet. The mask is imposed on the estimated light fields on the facet plane to reduce the error in the background. The parameter $\beta$ is used as the weight of the feedback ^1^, and the estimated complex fields on the facet are modified to

$$E_{n}^{'}=M\cdot E_{n}+ (1-M)[E^{'}(k-1)-\beta E_{n}] ,$$

where $E^{'}(k-1)$is the mean estimated complex field on the facet from the last iteration, and it is set as a zero-padded image in the first iteration. Therefore, the light fields are updated by the latest field in the masked region and the feedback from the background can be tuned by the weight $\beta$, which is set to 0.2. Then, the mean complex value $E^{'}(x,y)$of the two estimated fields on the facet plane is calculated by


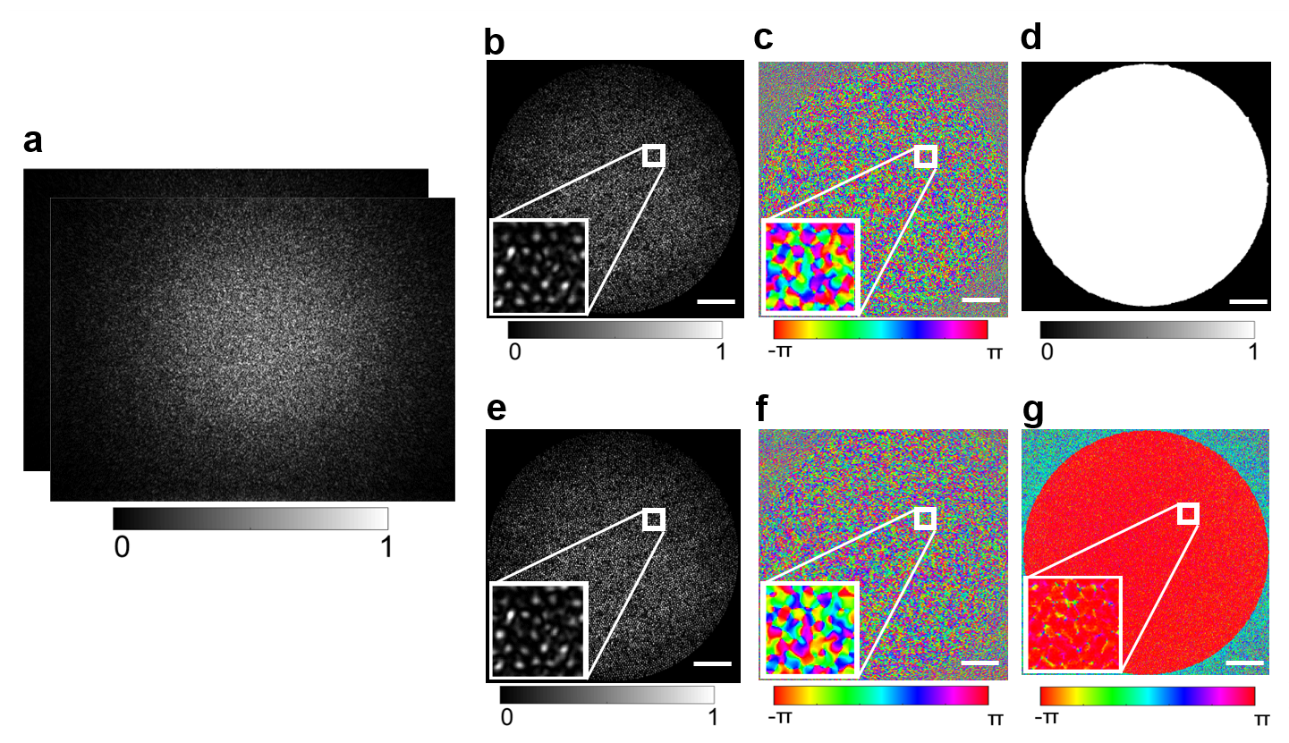


Fig. S2. Reconstruction of the reference phase distribution on the facet (a) Speckle images at 736μm and 800μm away from the facet. (b-c) Reconstructed (b) amplitude and (c) phase on the facet from the speckles. (d) Binary mask used as the weighted constraint. (e) Amplitude image of the fiber bundle facet. (f) Reconstructed phase on the facet from the off-axis holography. (g) Phase difference between (c) speckle reconstruction and (f) holography reconstruction. Scale bars 50μm.

$$E^{'}=\frac{1}{N}\sum_{n=1}^{N} E_{n}^{'},$$

$N$denotes the number of speckle images, and $N=2$ in this case. In the next step, the $E^{'}(x,y)$is propagated to the far-field located at $z_{1}$, $z_{2}$respectively

$$E_{F_{n}}^{'}=\text{iFFT}\{\text{FFT}\{E^{'}\} e^{ jz_{n}\sqrt{(2\pi/\lambda)^{2}- (k_{x}^{2}+k_{y}^{2})}}\}.$$

The new estimated phase on both far-field planes can be obtained from the updated complex field $E_{F_{n}}^{'}(x,y)$

Hence, the new complex fields in the far-field for the next iteration are updated by the estimated phase, and the original speckle images.

The algorithm runs iteratively until the complex light field on the facet is recovered correctly. The correlation coefficient between the reconstructed amplitude on the facet plane $\left|E^{'}(x,y)\right|$and the captured facet image is used as the figure of merit to characterize the performance of the algorithm. The reconstructed amplitude image on the facet after 2,200 iterations is demonstrated in Fig. S2b, which has a correlation coefficient of 0.96 compared with the amplitude image of the facet captured by the microscope (Fig. S2e). To characterize the fidelity of the phase reconstruction, the ground truth phase distribution on the facet (Fig. S2f) is measured with off-axis holography^5^ and the circular standard deviation ^6^ of the difference between the reconstructed phase and the ground truth in the facet region is used as the loss function. The phase deviation also converges when the amplitude correlation converges, and the tendencies of both criteria are very similar in the iteration process. Therefore, it is sufficient to use the amplitude correlation as the exit criteria to get an accurate phase reconstruction. The final reconstructed phase distribution on the facet is demonstrated in Fig. S2c, and the circular standard deviation of the phase difference to the ground truth is 0.41 rad in the facet region, which is 6.5% of 2π. Although there is still noticeable phase noise in the facet area, those noise mostly appears in the area of claddings (see Fig. S2g) which are not used to guide the light.


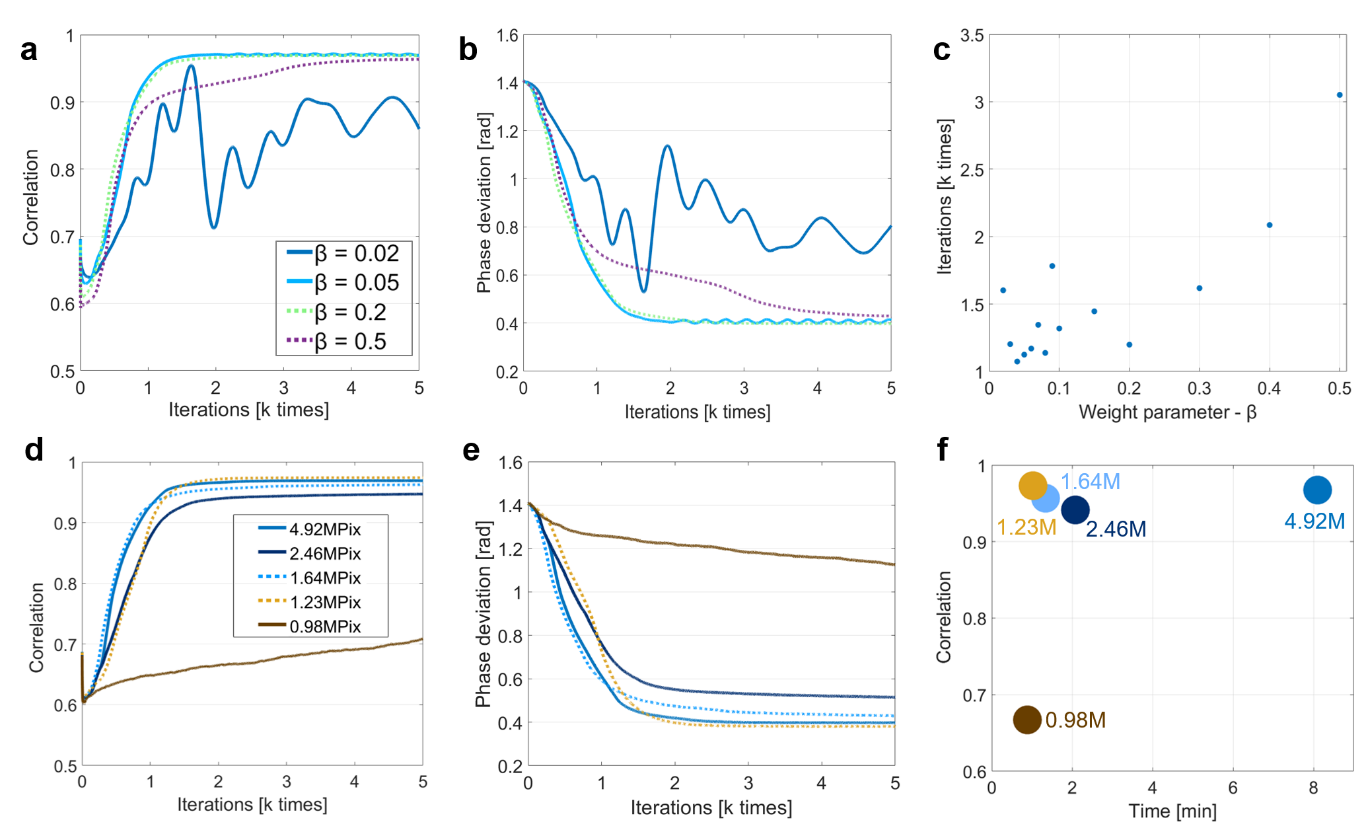


Fig. S3. (a-c) Comparison of the performance of the algorithm with different weight $\beta$. (a) The correlation coefficient between the reconstructed amplitude image and the captured image on the facet is used as the merit function. (b) The circular standard deviation of the phase difference between the reconstructed and holographic measured phase is used as the loss function. (c) The number of iterations to convergence with different weight parameter $\beta$. (d-f) Comparison of the performance of the algorithm with full camera pixels and 1/2, 1/3, 1/4, 1/5 sampled pixels. (d) The correlation coefficient is used as the merit function. (e) The circular standard deviation of the phase difference is used as the loss function. (f) Computation time and the correlation of the reconstruction for 2,200 iterations.

To characterize the performance of the reconstruction algorithm, the reconstruction process is performed and analyzed with different parameters. In Fig. S3, the correlation between the reconstructed amplitude and the captured amplitude image of the facet represents the fidelity of the reconstruction while the circular standard deviation of the phase difference between the reconstruction and holography measurement represents the error of the reconstruction. The amplitude correlation curve is highly correlated to phase deviation curves in different cases. The weight parameter $\beta$in Eq. 6 is used to tune the feedback of the estimated light field in the background region. As shown in Fig. S3a and b, the algorithm can hardly converge and oscillates randomly when $\beta\leq0.02$. For $0.03\leq\beta\leq0.06$, the algorithm can converge to a small range but there are still slight oscillations after the convergence. The algorithm converges smoothly for $\beta\geq0.07$. The required iteration time to achieve an amplitude correlation of 0.95 for different weight $\beta$ is demonstrated in Fig. S3c. It can be noticed that the convergence speed decreases when $\beta\geq0.3$, therefore, an optimal $\beta=0.2$ is chosen to achieve smooth and fast convergence.

The camera pixel size and the total number of pixels are both critical to achieving sufficient spatial sampling of the light field. According to the Nyquist–Shannon sampling theorem, to avoid aliasing and resolve the fiber cores with a diameter of $d_{\text{core}}$, the camera pixel size $a_{\text{cam}}$ should at least fulfill

$$a_{\text{cam}}<\frac{m\cdot d_{\text{core}}}{2},$$

where $m$ is the magnification ratio of the imaging system. In this work, the fiber core diameter is around 2μm and the magnification ratio of the imaging system is 10×. Therefore, the pixel size of the detection camera should be smaller than 10μm. The camera used in this work (UI-3482LE, IDS GmbH, Germany) has 4.92 million (2560×1920) pixels and the pixel size is 2.2μm.

To evaluate the influence of the quantity and size of camera pixels on the reconstruction performance. The speckle images are down-sampled to 2.46 million (1280×960), 1.64 million (853×640), 1.23 million (640×480), and 0.98 million (512×384) pixels. The pixel size set in the algorithm is also increased 2×, 3×, 4×, and 5× to keep the physical size of the image field the same. As shown in Fig. S3d and e, the performance of the algorithm is similar when the camera pixel size fulfills Eq. 11. When the speckle images are sampled to 512×384 pixels and the pixel size is increased to 11μm, the reconstruction can hardly converge. The computation time for 2,200 iterations and the correlation coefficient between the reconstructed amplitude image and the sampled ground truth image with the same pixel number is demonstrated in Fig. S3f. The computation time is decreased significantly with fewer pixels and the reconstruction fidelity remains high if Eq. 11 is fulfilled. It has to be noted that this result only denotes the performance of the reconstruction algorithm is robust when the camera sampling frequency is above the Nyquist frequency, and more camera pixels lead to higher spatial resolution.

**Spatial resolution measurement**


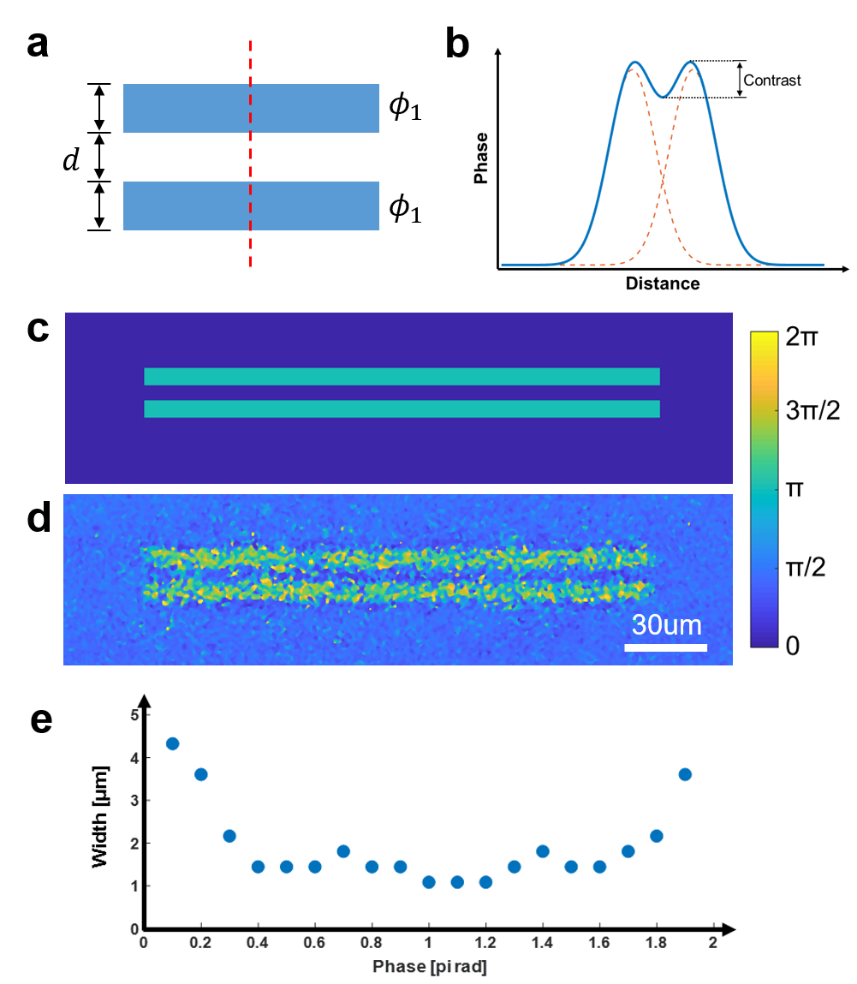


Fig. S4. The principle of quantifying the spatial resolution of the phase microendoscope. (a) A stripe pattern is used for the resolution test. The upper and bottom band shares the same width and phase, and the gap between the two bands has the same width of the lines. The red dashed line indicates the sampling direction of the phase profile. (b) The reconstructed phase profile in the marked direction. (c) The phase target displayed on the SLM. (d) Reconstructed phase image from the microendoscope. Scale bar 30μm. (e) The measured lateral resolution of the system for different phase values.

A program-controlled phase target shown in Fig. S4a is used to characterize the spatial resolution of the quantitative phase microendoscope. The length of the gap between the upper and bottom band is the same as the width of each band. An example of the phase distribution in the marked direction is shown in Fig. S4b. The contrast of the reconstructed phase profile is defined as the phase difference between the maximum and minimum phase value in the gap between the two bands. We define the contrast ratio (CR) as

where the is the maximum phase value. In ideal circumstances, the band is defined as resolvable when the CR is above the Rayleigh criterion, which is 27% here^7^.

To evaluate the resolution limit of our system at different phase values, this program-controlled phase target is displayed on a phase-only spatial light modulator (SLM; Pluto, Holoeye GmbH, Germany). As shown in Fig. S4c, the phase target is projected on the imaging plane of the MCF by a telescope system and the pixel size of the SLM is minified to 360nm. In each measurement process, the phase difference between the band and the background is set to a certain value. Firstly, the width of the band is set to one pixel, which corresponds to 360nm, and the corresponding speckle image is recorded on the detection side of the MCF. The phase image is thus reconstructed by the FAST method, and an example of the reconstructed phase image is shown in Fig. S4d. Hence, the CR of the reconstructed phase target is extracted from the corresponding phase profile sampled from the phase reconstruction. The width of the band is further increased in a step of one pixel until the CR of the reconstructed phase target is above the Rayleigh criterion. For instance, when the phase value of the band is set to π and the width of the band is two pixels (0.72μm), the CR of the reconstructed phase target is 24.2%. Then the width is increased to three pixels (1.08μm), and the CR is increased to 51.4%, which means the resolution limit of the system is 1.08μm for an ideal homogeneous object with a phase shift of π. We measured the resolution limit for phase value from 0.1π to 1.9π at a step of 0.1π, and the result is demonstrated in Fig. S4e and Fig. 4f.

**Quantitative phase measurement on a digital holographic microscope (DHM)**


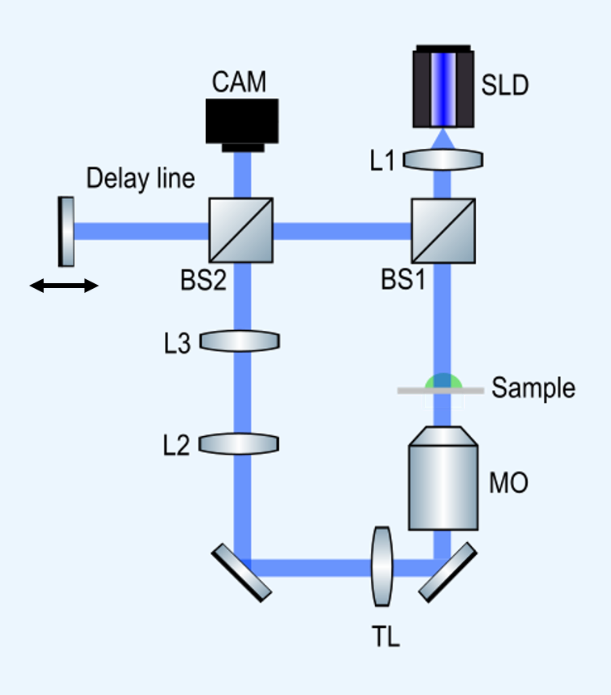


Fig. S5. Experimental setup of the digital holographic microscope. SLD, superluminescent diode; L1-L3, lenses; TL, tube lens; BS1, BS2, beamsplitters; MO, microscope objective; CAM, camera.

A DHM is implemented as a reference tool to measure the quantitative phase shift of the sample. The optical setup of the DHM is shown in Fig.S5. A superluminescent diode (SLD; 450nm; EXALOS AG, Switzerland), a low-coherence light source, is implemented to reduce the speckles in the phase reconstructions. A 40x microscope objective (0.65 NA; Plan Achromat Objective, Olympus) is used to image the PAAm beads shown in Fig. 5a in the main text. A 20× microscope objective (0.4 NA; Plan Achromat Objective, Olympus) is used to image the HeLa cells shown in Fig. 6d in the main text. The DHM is based on a Michelson interferometer. To match the optical path length of the object and reference beam, a mirror is mounted on a motorized stage for dynamic precise control of the path length of the reference beam. The second beamsplitter (BS2) is slightly tilted for off-axis holography, and the holographic reconstruction is based on the spatial filtering^5^ and angular spectrum method^8^. The reconstructed phase image is subtracted by the background to get the quantitative phase shift. The corresponding 3D OPD maps in Fig. 5a and 6d are calculated from the phase shift.

**Two-dimensional (2D) image correlation coefficient**

The 2D correlation coefficient is employed to characterize the fidelity of reconstructed amplitude images. Hence, for a normalized image$X(x,y)$ , the correlation coefficient (CC) between the reference image $Y(x,y)$ is expressed as

$$\text{CC}=\frac{\sum_{i}^{n} \left(X_{i}-\bar{X}\right)\left(Y_{i}-\bar{Y}\right)}{\left\{\sum_{i}^{n} \left(X_{i}-\bar{X}\right)^{2}\sum_{i}^{n} \left(Y_{i}-\bar{Y}\right)^{2}\right\}^{1/2}},$$

where $\bar{X}(x,y)$ and $\overline{Y}(x,y)$ is the mean value of the reconstructed image and the reference image respectively, and $n$ is the total number of pixels.

**Glass beads flow**

1mg glass beads (Dantec Dynamics, Denmark) are suspended in 0.5ml Dulbecco’s phosphate-buffered saline (DPBS) (Thermo Fisher, USA). Sizes of glass beads vary from 2μm to 20μm and the mean diameter is 10μm. The suspension is pumped into a microchannel constantly by a syringe. The height of the microchannel (µ-Slide Luer, ibidi GmbH, Germany) is 100μm and the channel volume is 25μl.


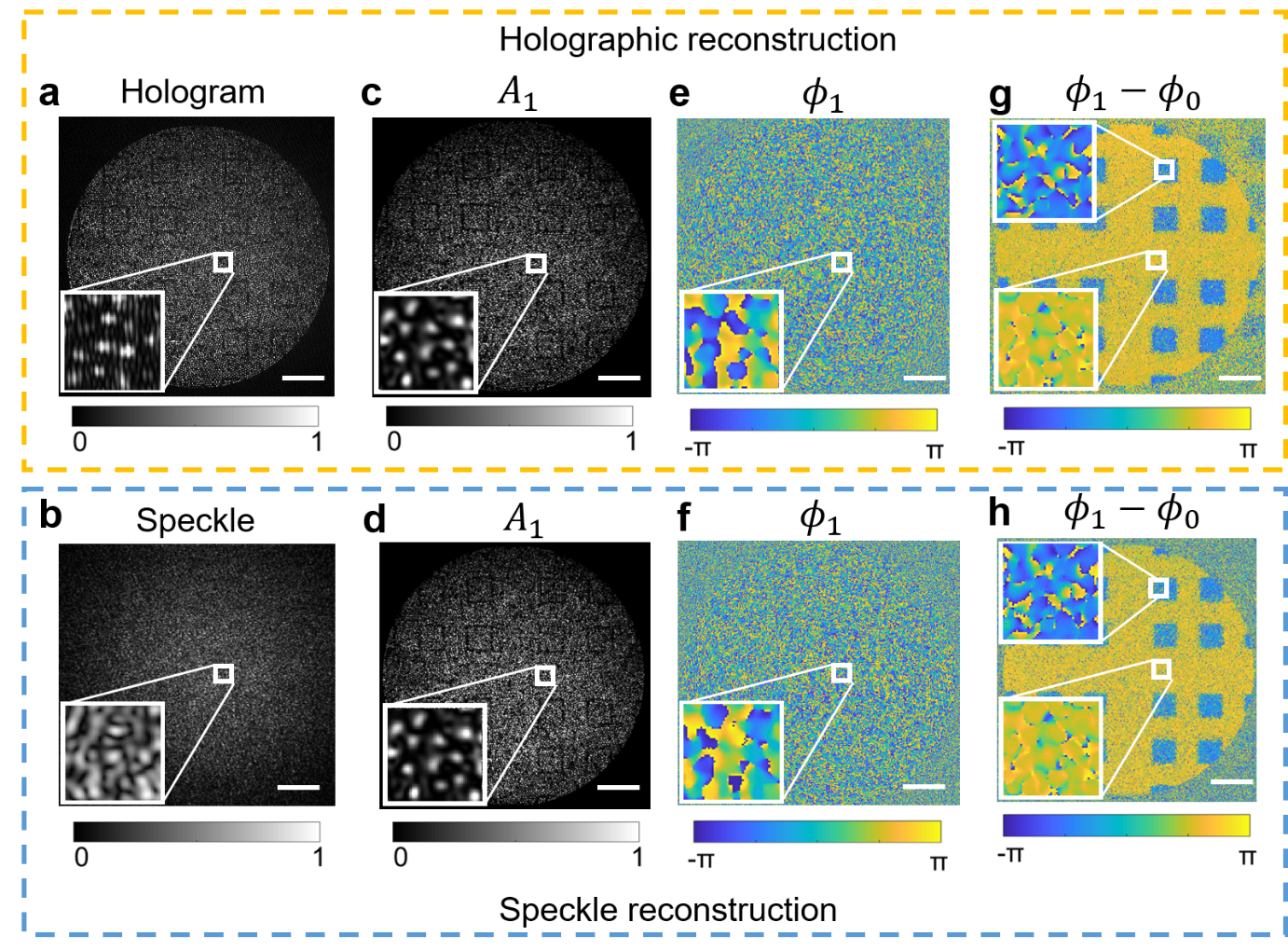


**Fig. S6. Comparison of off-axis holographic reconstruction and far-field speckle reconstruction.** (**a**) Off-axis hologram of a phase target captured at the facet on the detection side. (**b**) Far-field speckle image of the phase target captured on the far-field of the detection side. (**c-d**) Reconstructed amplitude image from the (**c**) hologram (**d**) speckle. (**e-f**) Reconstructed phase image from the (**e**) hologram (**f**) speckle. (**g-h**) Quantitative phase images of the target are retrieved from (**g**) holographic (**h**) speckle reconstruction. Scale bars 50μm.

Supplementary video V1.

Digital focusing of the multi-layer target in axial distances from 0 to 5mm away from the fiber facet at the measurement side. The top layer is digitally refocused at 1.26mm and the bottom layer is focused at 2.66mm away from the fiber facet.

Supplementary video V2.

Glass beads flowing through a microchannel are imaged by the lensless microendoscope at the measurement side. The speckle images, which are the system response shown on the left side of the video, are recorded by the detection camera at a video rate. The reconstructed video of the glass bead flow is shown on the right side of the video.

Supplementary video V3.

Reconstructed 3D optical path difference map of the phase target imaged through the quantitative phase microendoscope.

Supplementary video V4.

Reconstructed 3D optical path difference map of the HeLa cell in cytokinesis via the digital holographic microscope.

Supplementary video V5.

Reconstructed 3D optical path difference map of the HeLa cell in cytokinesis imaged through the quantitative phase microendoscope.

Supplementary information accompanies the manuscript on the Light: Science & Applications website (<http://www.nature.com/lsa>).

References

1. Fienup, J. R. Phase retrieval algorithms: a comparison. *Appl. Opt. Vol. 21, Issue 15, pp. 2758-2769* **21**, 2758–2769 (1982).

2. Rothe, S. *et al.* Benchmarking analysis of computer generated holograms for complex wavefront shaping using pixelated phase modulators. *Opt. Express* **29**, 37602 (2021).

3. Kogan, D. *et al.* Phase retrieval in multicore fiber bundles. *Opt. Lett.* **42**, 647 (2017).

4. Guo, C., Zhao, Y., Tan, J., Liu, S. & Liu, Z. Multi-distance phase retrieval with a weighted shrink-wrap constraint. *Opt. Lasers Eng.* **113**, 1–5 (2019).

5. Cuche, E., Marquet, P. & Depeursinge, C. Spatial filtering for zero-order and twin-image elimination in digital off-axis holography. *Appl. Opt.* **39**, 4070–4075 (2000).

6. Berens, P. CircStat: A MATLAB Toolbox for Circular Statistics. *J. Stat. Softw.* **31**, 1–21 (2009).

7. Cotte, Y., Toy, M. F., Shaffer, E., Pavillon, N. & Depeursinge, C. Sub-Rayleigh resolution by phase imaging. *Opt. Lett.* **35**, 2176 (2010).

8. Zhong, Z. *et al.* Automatic cross filtering for off-axis digital holographic microscopy. *Results Phys.* **16**, 102910 (2020).
